# Development of a Four‐Language Questionnaire to Investigate Environmental Risk Factors for the Development of Canine Atopic Dermatitis and to Monitor Disease Course and Progression

**DOI:** 10.1111/vde.70024

**Published:** 2025-09-01

**Authors:** Patricia Clara‐Maria Rhodius, Nina Fischer, Ana Rostaher, Franco Martini, Edwin Chapman, Sabrina Audergon, Stefan Hobi, Georg Lehner, Sylvie Wilhelm, Noëmi van Oordt, Claude Favrot, Malwina Kowalska

**Affiliations:** ^1^ Dermatology Department, Clinic for Small Animal Internal Medicine, Vetsuisse Faculty University of Zurich Zurich Switzerland; ^2^ Department of Veterinary Clinical Sciences City University of Hong Kong Hong Kong Hong Kong; ^3^ Kleintierpraxis Lehner ‐ Fachpraxis für Dermatologie Buch Germany; ^4^ Vet Dermatology GmbH Zurich Switzerland; ^5^ Department of Education University of Zurich Zurich Switzerland; ^6^ Ophthalmology Department, Clinic for Small Animal Internal Medicine, Vetsuisse Faculty University of Zurich Zurich Switzerland

**Keywords:** canine atopic dermatitis, dogs, environmental factors, questionnaire, reliability, validity

## Abstract

**Background:**

The chronic and multifactorial character of canine atopic dermatitis (cAD) often leads to poor disease control and treatment dissatisfaction. Environmental factors are likely to contribute to the disease development and may play a more important role than assumed previously. This opens new research directions that require an appropriate tool to obtain useful data from large representative study populations.

**Hypothesis/Objectives:**

A tool such as a questionnaire is suitable for obtaining high‐quality data to investigate the pathogenesis of cAD and monitor the disease.

**Materials and Methods:**

To assure the tool's validity and reliability, the development process of this four‐language questionnaire (original language German) included two pilot tests (with owners' interviews and questionnaire evaluation sheets), test–retest assessment, content validity evaluation and a structured translation and back‐translation into three languages (English, Italian and French).

**Results:**

The development process took place between June 2024 and December 2024. The preliminary questionnaire comprised 107 questions. The pilot tests (round 1 = four participants, round 2 = two participants) resulted in a revision of 31 questions and the deletion of three. The test–retest assessment revealed an Intraclass Correlation Coefficient of 0.80. The panel of (six) experts evaluated the questionnaire with a content validity index of 0.99. The translation and back‐translation process revealed that only minor adjustments were sufficient to guarantee the validity and reliability across languages.

**Conclusions and Clinical Relevance:**

The comprehensive development process ensures high validity and reliability of the questionnaire, indicating that such a process can not only positively impact the quality of the developed tool, but also create a reliable basis for the generation of accurate and less biased data.

## Graphical abstract

**Background:** The chronic and multifactorial character of canine atopic dermatitis (cAD) often leads to poor disease control and treatment dissatisfaction. Environmental factors are likely to contribute to the disease development and may play a more important role than assumed previously. This opens new research directions that require an appropriate tool to obtain useful data from large representative study populations. **Hypothesis/Objectives:** A tool such as a questionnaire is suitable for obtaining high‐quality data to investigate the pathogenesis of cAD and monitor the disease. **Conclusions and Clinical Relevance:** The comprehensive development process ensures high validity and reliability of the questionnaire, indicating that such a process can not only positively impact the quality of the developed tool, but also create a reliable basis for the generation of accurate and less biased data.

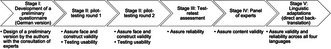

## ZUSAMMENFASSUNG

**Hintergrund:**

Der chronische und multifaktorielle Charakter der caninen atopischen Dermatitis (cAD) führt oft dazu, dass die Krankheit schlecht kontrolliert und die Behandlung unzufriedenstellend ist. Umweltfaktoren tragen mit großer Wahrscheinlichkeit zur Entwicklung der Krankheit bei und könnten eine größere Rolle spielen als bisher angenommen. Das eröffnet neue Forschungsrichtungen, die entsprechende Instrumente benötigen, um nützliche Daten aus großen repräsentativen Studienpopulationen zu erhalten.

**Hypothese/Ziele:**

Ein Instrument wie ein Fragebogen ist passend, um hochqualitative Daten zu erhalten, um die Pathogenese der cAD zu untersuchen und um die Krankheit zu überwachen.

**Materialien und Methoden:**

Um die Validität und die Verlässlichkeit dieses Instruments sicherzustellen, inkludierte der Entwicklungsprozess dieses Fragebogens in vier Sprachen (Originalsprache Deutsch) zwei Pilotstudien (mit Interviews und Fragebogen Evaluierungsbögen durch die BesitzerInnen), Test‐Retest Erfassung, inhaltliche Evaluierung der Validität, und eine strukturierte Übersetzung und Rückübersetzung in drei Sprachen (Englisch, Italienisch und Französisch).

**Ergebnisse:**

Der Entwicklungsprozess fand zwischen Juni 2024 und Dezember 2024 statt. Der vorläufige Fragebogen beinhaltete 107 Fragen. Der Pilot‐Test (Runde 1 = vier Teilnehmer, Runde 2 = zwei Teilnehmer) resultierte in einer Revision von 31 Fragen und der Entfernung dreier davon. Die Test‐Retest Erhebung zeigte einen Korrelationskoeffizienten von 0,80 innerhalb der Klassen. Die Gruppe der (sechs) Experten evaluierte den Fragebogen mit einem Inhaltsvaliditätsindex von 0,99. Die Übersetzung und der Rückübersetzungsprozess zeigte, dass nur geringfügige Anpassungen ausreichten, um die Validität und die Verlässlichkeit über die Sprachen hinweg zu gewährleisten.

**Schlussfolgerungen und klinische Bedeutung:**

Der umfassende Entwicklungsprozess sorgt für eine hohe Validität und Verlässlichkeit des Fragebogens, was darauf hinweist, dass ein solcher Prozess sich nicht nur positiv auf die Qualität des entwickelten Instruments auswirkt, sondern gleichzeitig eine verlässliche Basis zur Erschaffung genauer und weniger verzerrter Daten schafft.

## 摘要

**背景:**

犬特应性皮炎(cAD)具有慢性和多因素特征，这常常导致疾病控制不佳以及治疗满意度低。环境因素很可能促进该病的发生，并且其作用可能比以往认为的更为重要。这为新的研究方向打开了大门，而这些研究需要一种合适的工具，从大规模、具有代表性的研究人群中获取有用数据。

**研究假设/目标:**

问卷调查等工具适用于获取高质量数据，以研究cAD的发病机制并监测疾病。

**材料与方法:**

为保证工具的效度与可靠性，该四语种问卷(原始语言为德语)的开发过程包括两轮试点测试(与犬主访谈和问卷评估表)、测试–重测评估、内容效度评价，以及对三种语言(英语、意大利语和法语)的结构化翻译与回译。

**结果:**

开发过程发生在2024年6月至2024年12月之间。初步问卷共包含107个问题。试点测试(第一轮=4名参与者，第二轮=2名参与者)导致31个问题被修订，3个问题被删除。测试–重测评估显示组内相关系数为0.80。6名专家组成的小组对问卷的内容效度指数评估为0.99。翻译与回译过程中仅需进行少量调整，即可保证跨语言的有效与可靠性。

**结论与临床相关性:**

这一全面的开发过程确保了问卷的高效性与高可靠性，这表明该过程不仅能积极影响所开发工具的质量，还能为产生准确且偏倚较小的数据奠定可靠基础。

## Résumé

**Contexte:**

Le caractère chronique et multifactoriel de la dermatite atopique canine (DAC) entraîne souvent un mauvais contrôle de la maladie et une insatisfaction vis‐à‐vis du traitement. Les facteurs environnementaux sont susceptibles de contribuer au développement de la maladie et pourraient jouer un rôle plus important qu'on ne le pensait auparavant. Cela ouvre de nouvelles pistes de recherche qui nécessitent un outil approprié pour obtenir des données utiles à partir de populations d'étude représentatives importantes.

**Hypothèse/Objectifs:**

Un outil tel qu'un questionnaire est adapté pour obtenir des données de haute qualité afin d'étudier la pathogénie de la DAC et de surveiller la maladie.

**Matériels et méthodes:**

Afin de garantir la validité et la fiabilité de l'outil, le processus d'élaboration de ce questionnaire en quatre langues (langue originale: allemand) a compris deux tests pilotes (avec des entretiens avec les propriétaires et des fiches d'évaluation du questionnaire), une évaluation test‐retest, une évaluation de la validité du contenu et une traduction structurée et une retraduction en trois langues (anglais, italien et français).

**Résultats:**

Le processus d'élaboration s'est déroulé entre juin 2024 et décembre 2024. Le questionnaire préliminaire comprenait 107 questions. Les tests pilotes (1er tour = quatre participants, 2e tour = deux participants) ont abouti à la révision de 31 questions et à la suppression de trois autres. L'évaluation test‐retest a révélé un coefficient de corrélation intraclasse de 0,80. Le panel de (six) experts a évalué le questionnaire avec un indice de validité du contenu de 0,99. Le processus de traduction et de retraduction a révélé que seuls des ajustements mineurs étaient nécessaires pour garantir la validité et la fiabilité dans toutes les langues.

**Conclusions et pertinence clinique:**

Le processus de développement complet garantit une validité et une fiabilité élevées du questionnaire, ce qui indique qu'un tel processus peut non seulement avoir un impact positif sur la qualité de l'outil développé, mais aussi créer une base fiable pour la génération de données précises et moins biaisées.

## 要約

**背景:**

犬アトピー性皮膚炎(cAD)は慢性的かつ多因子的な性質を持ち、しばしば疾患のコントロール不良や治療の不満につながる。環境因子は疾患の発症に寄与している可能性が高く、従来考えられていた以上に重要な役割を果たしているかもしれない。これは、新たな研究の方向性を開き、大規模かつ代表性のある研究群から有用なデータを得るために適切なツールが必要となる。

**仮説/目的:**

アンケートのようなツールはcADの病因解明や疾患モニタリングのための高品質なデータの取得に適している。

**材料と方法:**

ツールの妥当性および信頼性を保証するために、4言語でのアンケート(原文はドイツ語)の開発過程には、2回のパイロットテスト(飼い主へのインタビューおよびアンケート評価用紙)、再テストによる評価、内容妥当性の評価、さらに英語・イタリア語・フランス語への体系化的な翻訳と逆翻訳を含んだ。

**結果:**

開発は2024年6月から2024年12月にかけて行われた。予備アンケートは107問で構成されていた。パイロットテスト(第1回=4名参加、第2回=2名参加)の結果、31問が修正され、3問が削除された。再テスト評価では、級内相関係数は0.80であった。(6名の)専門家によるパネル評価で、内容妥当性指数は0.99であった。翻訳および逆翻訳の過程では、各言語間での妥当性と信頼性を保証する上でごくわずかな調整で十分であることが確認された。

**結論と臨床的関連性:**

包括的な開発プロセスにより、アンケートの高い妥当性および信頼性が保証され、このようなプロセスは、開発されたツールの質を高めるだけでなく、正確で偏りの少ないデータを生成するための信頼できる基盤を構築することにもつながる。

## Resumo

**Contexto:**

O caráter crônico e multifatorial da dermatite atópica canina (DAc) frequentemente leva ao controle inadequado da doença e à insatisfação com o tratamento. Fatores ambientais provavelmente contribuem para o desenvolvimento da doença e podem desempenhar um papel mais importante do que se supunha anteriormente. Isso abre novas direções de pesquisa que exigem uma ferramenta apropriada para obter dados úteis de grandes populações de estudo representativas.

**Hipótese/Objetivos:**

Uma ferramenta como um questionário é adequada para obter dados de alta qualidade para investigar a patogênese da DAc e monitorar a doença.

**Materiais e Métodos:**

Para garantir a validade e a confiabilidade da ferramenta, o processo de desenvolvimento deste questionário em quatro idiomas (o idioma original é o alemão) incluiu dois testes piloto (com entrevistas com os tutores e folhas de avaliação do questionário), avaliação teste‐reteste, avaliação da validade de conteúdo e uma tradução e retrotradução estruturadas para três idiomas (inglês, italiano e francês).

**Resultados:**

O processo de desenvolvimento ocorreu entre junho de 2024 e dezembro de 2024. O questionário preliminar continha 107 perguntas. Os testes piloto (Rodada 1 = quatro participantes, Rodada 2 = dois participantes) resultaram na revisão de 31 questões e na exclusão de três. A avaliação teste‐reteste revelou um coeficiente de correlação intraclasse de 0,80. O painel de (seis) especialistas avaliou o questionário com um índice de validade de conteúdo de 0,99. O processo de tradução e retrotradução revelou que apenas pequenos ajustes foram suficientes para garantir a validade e a confiabilidade em todos os idiomas.

**Conclusões e relevância clínica:**

O processo abrangente de desenvolvimento garante alta validade e confiabilidade do questionário, indicando que tal processo pode não apenas impactar positivamente a qualidade da ferramenta desenvolvida, mas também criar uma base confiável para a geração de dados precisos e menos tendenciosos.

## RESUMEN

**Introduccion:**

El carácter crónico y multifactorial de la dermatitis atópica canina (cAD) suele provocar un control deficiente de la enfermedad y la insatisfacción con el tratamiento. Es probable que los factores ambientales contribuyan al desarrollo de la enfermedad y desempeñen un papel más importante de lo que se creía anteriormente. Esto abre nuevas líneas de investigación que requieren una herramienta adecuada para obtener datos útiles de poblaciones de estudio representativas y de gran tamaño.

**Hipótesis/Objetivos:**

Una herramienta como un cuestionario es adecuada para obtener datos de alta calidad que permitan investigar la patogénesis de la cAD y monitorizar la enfermedad.

**Materiales y métodos:**

Para garantizar la validez y fiabilidad de la herramienta, el proceso de desarrollo de este cuestionario en cuatro idiomas (idioma original: alemán) incluyó dos pruebas piloto (con entrevistas a propietarios y hojas de evaluación del cuestionario), una evaluación test‐retest, una evaluación de la validez de contenido y una traducción estructurada y una retrotraducción a tres idiomas (inglés, italiano y francés).

**Resultados:**

El proceso de desarrollo se llevó a cabo entre junio y diciembre de 2024. El cuestionario preliminar constaba de 107 preguntas. Las pruebas piloto (Ronda 1 = cuatro participantes, Ronda 2 = dos participantes) resultaron en la revisión de 31 preguntas y la eliminación de tres. La evaluación test‐retest reveló un coeficiente de correlación intraclase de 0,80. El panel de seis expertos evaluó el cuestionario con un índice de validez de contenido de 0,99. El proceso de traducción y retrotraducción reveló que solo pequeños ajustes fueron suficientes para garantizar la validez y la fiabilidad en todos los idiomas.

**Conclusiones y relevancia clínica:**

Un exhaustivo proceso de desarrollo garantiza una alta validez y fiabilidad del cuestionario, lo que indica que dicho proceso no solo puede influir positivamente en la calidad de la herramienta desarrollada, sino que también crea una base fiable para la generación de datos precisos y menos sesgados.

## 1 Introduction

An assumed hypothesis by veterinary dermatology clinicians is that environmental factors play a prominent role in the development and progression of canine atopic dermatitis (cAD) [1]. These factors, particularly during early life stages, including prenatal exposure, potentially trigger inflammation through activation of the innate immune system [1], which may favour the potential development of an allergy. This shift in the conceptualisation of cAD pathogenesis opens new research directions focused on investigating environmental factors.

Identifying specific environmental factors responsible for cAD development and progression may help disease prevention and management. Being able to generate this knowledge is pivotal for developing individualised therapeutic interventions. Understanding the timing and nature of these factors could also enhance the precision of future clinical and epidemiological studies, with the thought that data collection can become more precisely focused. It could enable more detailed insights by allowing for a more accurate alignment with the underlying factors, facilitating the generation of more targeted information.

The management of cAD typically requires complex, long‐term therapeutic approaches and high owner compliance. Treatment strategies often involve multiple concurrent therapies or multiple changes in the therapy regime over a long period of time [2]. However, therapeutic success depends heavily on the consistency and reliability of the management and treatment strategies, which many owners find difficult or frustrating. Referral to veterinary dermatologists is often driven by severe clinical signs, treatment failures or the desire for advanced diagnostics [2]. This, in turn, supports the theory that many owners experience frustration over time and feel the need for better disease management.

In order to support clinical care and research, the development of a standardised tool is required. Such a tool could be used to ensure both the generation of the necessary data on environmental risk factors and the regular monitoring of the disease progression and response to treatment.

Questionnaires offer a practical method for collecting longitudinal data in clinical research from large‐scale study populations. Their ease of use and accessibility, facilitated by digital platforms, make them valuable tools for gathering insights from diverse populations [3]. However, the development of effective questionnaires requires careful design to ensure data quality [3]. Additionally, the subjectivity of respondents highlights the need to assess both the questionnaires' validity and reliability before broader implementation [4].

Validity in this context means that the developed questionnaire accurately measures the intended constructs; for example, all relevant aspects of the measured concept are adequately represented. Reliability is a measure of consistency across repeated administrations [5]. These properties are critical for generating comparable and co‐interpretable data [5]. Without them, results may be skewed by bias through subjective perceptions or inconsistency.

The focus of this study in terms of validity is on providing the questionnaire's face, content and construct validity, three of the different types that validity also includes criterion, concurrent, external and internal validity [5]. The focus on the face, content and construct was considered justified as they were deemed most relevant to achieving the set goals, and the inclusion of all validity types would have been beyond the scope of the study.

Face validity is used to assess how, for example, individual questions are perceived by the respondents [6]. Construct validity assesses how well a study's measurements (in this case the questionnaire) actually reflect the theoretical concepts they are intended to represent [7]. An important criterion for any form of validity assessment, including content validity, is that it establishes the construct validity of the underlying concept being measured [8].

In order to ensure the validity and reliability of the questionnaire to this extent, the study comprises the following five stages: development of a preliminary questionnaire (I), two rounds of pilot‐testing (II), the assessment of the test–retest reliability (III), a review via a panel of experts (IV) and a structured translation and back‐translation process (V).

The questionnaire developed within this study (see Appendix S4) is currently being used to assess two populations: owners of dogs diagnosed with cAD (study population) and owners of those without dermatological conditions (control group), all sourced from the corresponding research animal hospital.

The aim of this study was to develop a questionnaire that ensures a high level of validity and reliability, which can be used to investigate environmental risk factors for the development of cAD, as well as to monitor the disease course and progression. In this publication, we aimed to share our experience and provide insights into the methodology underlying the development process.

## 2 Materials and Methods

### 2.1 Ethical Approval

The study received an affirmative evaluation from the corresponding ethics committee. All participants, including dog owners and experts involved in the development and judgement process of the questionnaire, gave their consent to participate.

### 2.2 Questionnaire Development Stages and Preregistration

**FIGURE 1:**
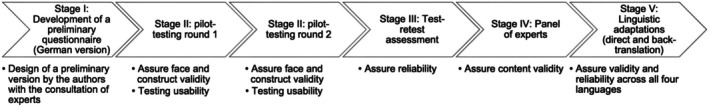
Canine atopic dermatitis questionnaire development stages.

The study protocol was preregistered on Open Science Framework (OSF) [9] on 3 September 2024 and is available under DOI: https://doi.org/10.17605/OSF.IO/6TN4R. The questionnaire development took place between June 2024 and December 2024. It included the development of a preliminary questionnaire (Stage I), two rounds of pilot testing (Stage II), the assessment of the test–retest reliability (Stage III), a review via a panel of experts (Stage IV) and a structured translation and back translation process (Stage V), all illustrated in Figure 1.

#### 2.2.1 Stage I: Development of Preliminary Questionnaire (German Version)

A preliminary version of the questionnaire was designed by the researching authors (P.C.‐M.R., C.F. and M.K.) on the grounds of their knowledge about canine atopic dermatitis and the consultation of diplomates of the European College of Veterinary Dermatology (Dipl. ECVD). These experts provided information about potential risk factors for the development of cAD to the authors via email. The researching authors also took into account which data about dogs with cAD are currently not collected (yet necessary) at the corresponding animal research hospital via the electronic health record system. Additionally, two veterinary epidemiologists and qualitative researchers were consulted on how a preliminary version of the questionnaire could best be realised without being dominated exclusively by the veterinary background. The preliminary questionnaire consisted of 12 question categories that contain a total of 107 questions: (1) general information (e.g., date of birth and signalment), (2) family (information about potential allergies among relatives), (3) atopic dermatitis (e.g., disease start, current clinical signs, allergy diagnostic and current treatment), (4) associated diseases (e.g., ear infections and pyoderma), (5) general hygiene (e.g., information about deworming, ectoparasite prophylaxis), (6) allergology, (7) the environment the dog was born in (8) and currently lives in, (9) feeding, (10) medical history (regarding any other diseases than allergies), (11) previous treatment and (12) skin and fur.

#### 2.2.2 Stage II: Pilot‐Testing Rounds 1 and 2 (Face and Construct Validity)

In brief, pilot‐testing is a key phase for enhancing the reliability, validity and feasibility of questionnaires [10]. It helps to identify ambiguous or ineffective questions. This allows researchers to refine the questionnaire to ensure that it is comprehensive and appropriate. Pilot‐testing should involve a sample size sufficiently large to assess performance yet sufficiently manageable to allow detailed feedback [11]. Put together, pilot‐testing involves administering the preliminary questionnaire to a representative sample with broad characteristics and collecting their feedback through appropriate tools, for example, questionnaire evaluation sheets.

The intended goals of the pilot‐testing rounds were to assess the questionnaire's face and construct validity. Another key consideration of pilot‐testing was the utility, specifically how practical the questionnaire is for application in field settings [12]. This includes, for example, how easily the questionnaire is accessible for the participants (online survey Appendix S1) and how well it can be answered on a computer.

The participants of the pilot‐testing rounds were six owners (aged from mid‐20s to mid‐70s, with different levels of education [academics and non‐academics] and of diverse gender) of dogs with cAD (patients of the corresponding research animal hospital). In September 2024, the clinical schedule was reviewed, and owners were invited to participate via email 2 days in advance of their scheduled appointment; nonrespondents were invited by one of the authors (P.C.‐M.R.) in person on the day of their dogs' appointment.

In pilot‐testing round 1 four owners participated. The owners were invited on site to complete the preliminary questionnaire online using the program limesurvey [13] on a laptop provided by one of the authors (P.C.‐M.R.). While being observed by this author, they were prompted to provide comments whenever they had doubts about questions. Their comments were immediately collected and written down by the observing author. Afterwords, they were asked to fill in a questionnaire evaluation sheet, which comprised six closed‐ended questions (Table 1). The questions could be answered with one of the following options: ‘agree’, ‘rather agree’, ‘rather disagree’ and ‘disagree’. Also, the time duration the participants needed to fill out the questionnaire was documented.

**TABLE 1:** Questions from the questionnaire evaluation sheets to assess the face and construct validity of the preliminary canine atopic dermatitis questionnaire (pilot‐testing rounds 1 and 2).

Question no.	Questions: Round 1	Questions: Round 2
1	The questions are clearly formulated, and I had no difficulties in understanding them	How would you evaluate the questions in terms of wording and comprehensibility?
2	I consider the questions useful for gathering valuable information about my dog's health	How would you rate the relevance of the questions? Are the questions capable of gathering important information about your dog's state of health?
3	I think the survey is clearly structured and easily comprehensible	How would you evaluate the survey in terms of structure and comprehensibility?
4	It was simple for me to complete the questionnaire on the computer	How would you describe the process of completing the questionnaire on the computer?
5	I had no technical problems while completing the questionnaire	Did you have any technical problems while completing the questionnaire? If yes which?
6	The time it took me to complete the questionnaire was appropriate for me	How did you rate the time it took you to complete the questionnaire?

In pilot‐testing round 2, two other owners participated. The process was the same except that the formulation of the evaluation questions was changed to open‐ended questions (Table 1). The closed or so‐called ‘forced‐choice’ style of asking questions is a proven survey format, which allows for easy scoring [14]. However, it was decided to change the format because open‐ended questions provide the opportunity for free responses, which is a more suitable format for gathering opinions [15]. The potential for bias is minimised by not suggesting a particular reply [16].

The participants' comments and the evaluation sheets were analysed by the researching authors to decide which questions needed to be revised, which questions were missing or needed to be deleted to demonstrate adequate face and construct validity, and whether there were any improvements that could be made to the implementation of the questionnaire (including technical issues) to meet the participants' needs.

#### 2.2.3 Stage III: Test–Retest Assessment (Reliability)

In brief, the test–retest assessment is used to check the questionnaires' reliability [12]. It is facilitated by comparing results from the same respondents across two time points [12, 17]. The interval between tests must be carefully chosen. It should be long enough to prevent a memory effect and short enough to avoid genuine changes in responses [18].

As one round of the test–retest assessment was considered to be sufficient, the four participants from pilot‐testing round 1 were asked to complete the same preliminary questionnaire again after 4 days, this time remotely. The answers were analysed statistically using the Intraclass Correlation Coefficient (ICC) to assess the level of reliability [18]. For a proper use of the ICC, a reasonable choice of the selected variant is needed [19]. Hence, the variant of the ICC used for the test–retest reliability was two‐way random effect. The ICC was calculated with the irr package in the statistical software R studio [19]. The ICC is interpreted as described by Koo and Li [20].

#### 2.2.4 Stage IV: Panel of Experts (Content Validity)

The Lynn method [21], where the content validity is evaluated via a panel of experts, was chosen to assess the content validity of the developed questionnaire. Six veterinary clinicians (panel of experts) received the questionnaire via email together with an instruction on how to evaluate the content validity of each individual question as well as the questionnaire itself (Appendix S1).

In order to quantify the content validity, the Content Validity Index (CVI) of each question as well as of the whole questionnaire needs to be assessed [21]. In brief, the CVI was calculated with the use of a four‐point ordinal rating scale (Table 2) and a formula presented in Appendix S1. The CVI is derived by dividing the number of experts (*n*) who rated the question as content‐valid by the total number (*N*) of experts who are included in the judgement process (*n/N*) [21]. The CVI of the entire questionnaire results in the number of questions judged as content‐valid divided by the total number of questions [21].

**TABLE 2:** Four‐point rating scale for the assessment of the content validity via the panel of experts.

Degrees of relevance	
1	The question is not relevant to achieve the set goal
2	The question is somewhat relevant to achieve the set goal (the question would need to be changed to be more relevant)
3	The question is relevant to achieve the set objective (the question is applicable or requires only minor adjustment)
4	The question is very relevant to achieve the set goal

A question was considered to be content‐valid if experts rated it with a Number 3 or 4. According to Lynn, a question is still evaluated as content‐valid even if one expert has ranked it with a number < 3 when a minimum of six experts are included in the judgement process [21].

According to the methodology, it is suggested that if the individual questions as well as the entire questionnaire have been assessed as content‐valid by the experts, then no further revisions are required [22]. As the aim of the researching authors was to obtain sufficient content validity for each individual question as well as for the questionnaire itself, they decided that any question with an insufficient content validity assessment would be revised based on the experts' feedback.

#### 2.2.5 Stage V: Linguistic Adaptations (Direct and Back‐Translation)

Given Switzerland's multilingual regions [23] and the potential for international research, linguistic adaptation needed to be an additional step in the development process. These included a structured direct and back‐translation process as well as proofreading through native speakers. The original version of the questionnaire, written in German by a native speaker (P.C.‐M.R.), was subsequently translated with the online translator deepl (Deepl SE) [24] into English, Italian and French. The three deepl translated versions were proofread by veterinarians who are native speakers and fluent in German (proofreaders). The corrected deepl versions were subsequently retranslated into German by the proofreaders (E.C., F.M. and C.F.) following the procedure of a back‐translation process [25, 26]. The back‐translated versions were sent back to one of the authors (P.C.‐M.R.) as word documents (v16.94 [25020927]; Microsoft), who then compared them with the original German version regarding every individual question and answer option. If there were any differences in sentence structure, grammar or meaningfulness (linguistic specifications), they were noted and then discussed with the corresponding proofreader to decide whether the corresponding question or answer option needed to be revised regarding the aforementioned linguistic specifications of sentence structure, grammar and meaningfulness.

### 2.3 First Implementation of the Questionnaire and Further Outlook

The online survey tool limesurvey (LimeSurvey GmbH) [13] was used for a suitable integration of the questionnaire so that participants could work on it remotely. All owners in the system of the corresponding research animal hospital who agreed to be contacted about clinical veterinary studies since the year 2020 received the questionnaire via email (4874 owners of dogs without cAD and 589 owners of dogs with cAD). It was decided by the authors that participation reminders for the survey would be distributed twice, initially with a 4‐week interval, followed by a 3‐week interval. The long‐term goal was to carry out the questionnaire with owners of dogs with cAD on a regular basis.

### 2.4 Statistical Methods

The statistical methods used to evaluate the results were, as described above, the calculation of the ICC using R studio (RStudio Team) [27] and the assessment of the CVI using the Lynn method [21].

## 3 Results

**FIGURE 2:**
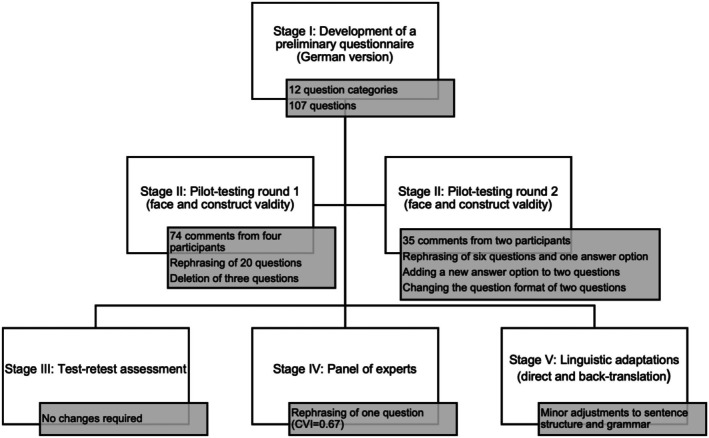
Number of changes to the preliminary canine atopic dermatitis questionnaire based on the results of the development stages.

The adaptations to the preliminary questionnaire resulting from the individual development stages are illustrated in Figure 2.

### 3.1 Stage II: Pilot‐Testing Rounds 1 and 2 (Face and Construct Validity)

In pilot‐testing round 1, a total of 47 verbal comments from four participants were obtained. The average number of comments on the questionnaire per participant was 12 (minimum nine to maximum 16). The number of comments on each question is visualised in Appendix S2.

Four of four participants answered the Questions 2, 3, 4 and 6 (Table 1) from the questionnaire evaluation sheet with ‘agree’. Question 1 received the feedback ‘agree’ from three participants, and one person answered with ‘rather agree’. Question 5, related to technical issues, was answered with ‘agree’ by two of four participants, and with ‘rather agree’ and ‘disagree’ by one of four each. The results are illustrated in Table 3.

**TABLE 3:** Answers to the closed‐ended evaluation questions from pilot‐testing round 1.

Question no.	Questions: Round 1	Respondent				Overall, of rather agree/disagree and agree answers
		I	II	III	IV	*n*/*N* (%)
1	The questions are clearly formulated, and I had no difficulties in understanding them	Agree	Agree	Rather agree	Agree	1/4 (75)
2	I consider the questions useful for gathering valuable information about my dog's health	Agree	Agree	Agree	Agree	0/4 (100)
3	I think the survey is clearly structured and easily comprehensible	Agree	Agree	Agree	Agree	0/4 (100)
4	It was simple for me to complete the questionnaire on the computer	Agree	Agree	Agree	Agree	0/4 (100)
5	I had no technical problems while completing the questionnaire	Disagree	Agree	Agree	Rather agree	2/2 (50)
6	The time it took me to complete the questionnaire was appropriate for me	Agree	Agree	Agree	Agree	0/4 (100)

Based on the participants' verbal comments, indicating that some questions were not interpreted as intended by the authors, 20 questions were rephrased regarding sentence structure and/or wording. One example is shown in Appendix S3. Three of the 107 questions were completely deleted from the preliminary questionnaire as the participants did not answer as expected by the authors. Thus, the questions would not have provided the information that was intended to be investigated.

In pilot‐testing round 2, 35 verbal comments on the questionnaire were submitted by two participants (Participant 1 = 22 comments, Participant 2 = 13 comments). The number of comments on each question also is visualised in Appendix S2. The evaluation of the answers to the six questions from Table 1 (round 2) resulted in the impression of a positive assessment from the participants towards the questionnaire.

After pilot‐testing round 2, six questions were rephrased for better comprehension, a new answer option to two questions was added for more detailed information, and a possible answer for one question was rephrased. Furthermore, two questions were changed regarding their format from multiple to single‐choice, as several possible answers might have led to an overly wide range of results. No question was deleted.

The average time to complete the questionnaire was 26 min. Of all six pilot‐testing participants, the fastest answered the questionnaire in 16 min (Participant 2 from pilot‐testing round 2), and the slowest in 30 min (Participant 4 from pilot‐testing round 1). However, it should be mentioned that the number of questions to be answered is not the same for every participant; some questions are only displayed if a previous question has been answered coherently.

The authors believe that thanks to the above‐mentioned process, the face and construct validity of the cAD questionnaire are ensured.

### 3.2 Stage III: Test–Retest Assessment (Reliability)

Two participants completed the repeatedly conducted questionnaire as required (within 4 days). One person re‐answered after 5 days and the other after 7 days. The test–retest reliability measure provided an ICC of 0.80, indicating good reliability [20].

### 3.3 Stage IV: Panel of Experts (Content Validity)

**FIGURE 3:**
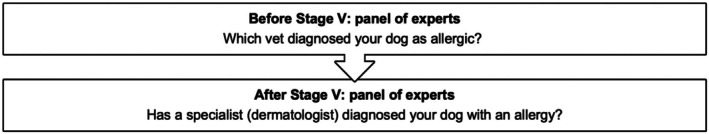
Rephrasing of the question with a content validity index of 0.67 to reach a sufficient level of content validity.

All experts rated 82 questions as content‐valid (CVI of 1.00). Twenty‐one questions were regarded as content‐valid by five experts (CVI of 0.83). One question was rated as not content‐valid, as only four of six experts rated it with a number > 2 (CVI of 0.67). Two experts provided the feedback that the phrasing of this question needed to be more restrictive. Therefore, this question was revised and rephrased accordingly to the experts' feedback (Figure 3). The assessment of the content validity of the whole questionnaire resulted in a CVI of 0.99.

### 3.4 Stage V: Linguistic Adaptations (Direct and Back‐Translation)

The native speakers judged the phrasing and meanings of the deepl translations (Deepl SE) [24] as being high quality. In comparison with the original, the retranslated versions only showed differences in sentence structure (three questions from the English version) and grammar (two questions from the Italian version), as meaningfulness was judged to be preserved across all questions and answer options. As the focus was on maintaining equivalent meaningfulness among the translations, only minor adjustments to the sentence structure and grammar were required. Therefore, five questions in the translated versions were adjusted. Hence, it was determined that validity and reliability were upheld across all four languages.

### 3.5 First Implementation of the Questionnaire and Further Outlook

A total of 31% (1703) responses were received from 5463 contacted owners (4874 with dogs diagnosed with cAD, 589 with presumed nonallergic dogs). Of these 1703, 805 (of 47%) questionnaires were complete and 898 (53%) were incomplete. There are several reasons for incomplete questionnaires. Some owners sent an email after partially completing the questionnaire and stated that they had intended to finish, yet unfortunately forgot to do so before the survey deadline. Others stated that they wanted to take a look at the questionnaire out of curiosity, yet did not want to answer the questions seriously for various reasons (e.g., death of their dog). Additionally, some respondents skipped individual questions, and some questionnaires were left incomplete for unknown reasons. Among contacted owners, 41% (241 of 589) of those with allergic dogs responded (including 222 complete questionnaires) and 13% (633 of 4874) of those with presumed nonallergic dogs responded (including 583 complete questionnaires).

The questionnaire was accessible for a duration of 8.5 weeks. The results of the first implementation of the questionnaire are currently being analysed. Targeted at achieving the set long‐term goal, the regular implementation of the questionnaire for the owners of dogs with cAD is presently in the planning phase.

## 4 Discussion

The main focus of the present study was to demonstrate the development process of a questionnaire to the veterinary dermatology research community. The questionnaire was specifically developed to enhance the understanding and management of cAD, while assuring its high validity and reliability, as well as obtaining high‐quality data. The described methods draw primarily from epidemiological and sociological research. The key challenge of this study was in integrating clinical knowledge and expectations of clinical researchers with epidemiological and sociological methods.

Both pilot‐testing rounds showed that there is a difference in the comprehension and interpretation of formulations between people with professional experience (questionnaire authors) and people without specialised knowledge (dog owners who filled in the questionnaire). This discrepancy highlighted the necessity of pilot‐testing to ensure that the questionnaire aligns with both the developers' intentions and the participants' needs. The results of pilot‐testing round 1 in particular showed that questions did not appear as clear to the owners as the authors had intended and therefore could not be answered as originally assumed. The pilot‐testing rounds were essential for identifying and addressing differences in understanding.

Initially, some authors and consulted experts raised concerns about the questionnaire's scope and its estimated completion time of 30 min, fearing it might discourage participation. However, pilot‐testing provided reassuring results: Five of six participants completed the questionnaire in less time and provided positive feedback regarding its length. As a result, no shortening was necessary. Pilot‐testing allowed the authors to verify initial concerns and preserve comprehensive data collection. Incorporating owners' perspectives in questionnaire development ensures that the content reflects what they deem important about the disease, thereby enhancing the relevance and validity of the data collected. This owner‐centred approach, analogous to patient‐centred practices in human medicine, is particularly critical in veterinary contexts, where owners serve as proxies for the patient, highlighting once more the importance of including the patient owners in the development process of a questionnaire.

Expert feedback is a critical component in the development of questionnaires, particularly for ensuring content validity. It helped to identify potential weaknesses and ambiguities that were not apparent to the authors. Notably, one question with a CVI of 0.67 was revised not based on the researching author's judgement, but instead guided by the feedback of independent experts. This process highlights the value of expert consultation in enhancing questionnaire design.

It can be presumed that translating into three languages is a complex process, particularly if the focus is on providing validity and reliability across languages. Because all communicative processes are primarily shaped by the cultural context in which they occur, along with the communicative behaviours of the individuals involved [28], misunderstandings or ambiguity in the meaning of words can arise when a concept developed in one culture is translated for its usage in another [25]. As the German version was developed by a native speaker, the cultural context of the language was inherently considered. Given that the initial translations were generated using deepl (Deepl SE) [24], the cultural context may not have been adequate. Therefore, the corresponding proofreaders were of Swiss (German Swiss and Italian Swiss), English and French nationality. Notwithstanding this, in the described case only errors regarding sentence structure and grammar were detected. The authors believe that a back‐translation process should be implemented in every multilingual questionnaire. Equivalent linguistic adaptations between the versions can not only simplify the completion of the questionnaire, but also facilitate the interpretation of the collected multilingual data.

The authors would like to emphasise that a questionnaire such as the one developed in the present study seems to be very desirable among the dermatology community, as several veterinary clinicians had already expressed their interest in it. It is possible that the current absence of a comparable tool is a consequence of the challenges of both a lengthy development process requiring several months of time and the need for collaboration between multiple experts and researchers.

The study has some limitations. First, the change in question style between the pilot‐testing rounds may have influenced responses, yet similar results across formats imply minimal impact. Second, some participants exceeded the 4‐day interval, which may have caused recall bias; however, the ICC and the stable question content mitigate this issue.

A third limitation is that the sample size of the pilot‐testing was rather small, with a total of six participants, a number which could be insufficient to draw a reasonable conclusion about the validity and reliability of a questionnaire that is intended to be administered to a significantly larger number of people. However, this sample size was chosen based on the experts' recommendations, based on the fact that the questions have stable content and that the respondents (especially considering the long‐term goal) are mainly owners of dogs with cAD who have either been clients at the research animal hospital for a long time or for whom long‐term care of the dogs is planned. Also, care was taken when selecting the people for the pilot‐testing to ensure that there was demographic variance (e.g., age, highest educational qualification, gender) in order to check and ensure that the questionnaire met the needs of all respondents as far as possible.

Regarding the results of the questionnaire pilot‐testing and positive participant feedback, it can be assumed that broader use of it will similarly impact the investigation and monitoring of cAD. Long‐term implementation will show the extent to which the management of cAD will actually benefit from the use of the questionnaire by owners. Lastly, the response rates may depend on owner motivation, treatment success and access to or familiarity with digital tools, as the survey is only available online.

## 5 Conclusions

Any questionnaire development process that aims at a high level of validity and reliability may be time‐ and resource‐consuming. However, engaging dog owners and domain knowledge experts, as well as specialists from epidemiology and social science in the development process can not only positively impact the quality of the developed tool, but also hopefully generate more accurate data.

## Author Contributions

**Nina Fischer:** methodology (equal), writing – review and editing (lead). **Patricia Clara‐Maria Rhodius:** conceptualization, investigation, writing – original draft, writing – review and editing, methodology, validation, visualization, formal analysis, data curation. **Franco Martini:** methodology (equal), writing – review and editing (lead). **Sabrina Audergon:** methodology (equal), writing – review and editing (lead). **Edwin Chapman:** methodology (equal), writing – review and editing (lead). **Claude Favrot:** conceptualization, investigation, funding acquisition, writing – review and editing, validation, methodology, supervision, project administration. **Sylvie Wilhelm:** methodology (equal), writing – review and editing (lead). **Stefan Hobi:** methodology (equal), writing – review and editing (lead). **Georg Lehner:** methodology (equal), writing – review and editing (lead). **Ana Rostaher:** methodology (equal), writing – review and editing (lead). **Malwina Kowalska:** conceptualization, investigation, writing – review and editing, validation, methodology, supervision, data curation, formal analysis. **Noëmi van Oordt:** methodology (equal), writing – review and editing (lead).

## Conflicts of Interest

The authors declare no conflicts of interest.

## Supporting information


**Appendices S1–S4:** vde70024‐sup‐0001‐AppendixS1‐S4.docx.

## Data Availability

The data that support the findings of this study are available from the corresponding author upon reasonable request.
