# Supplementary material for: Development of a Four‐Language Questionnaire to Investigate Environmental Risk Factors for the Development of Canine Atopic Dermatitis and to Monitor Disease Course and Progression
Source: Vet Dermatol. 2025 Sep 1;37(1):34–44. doi: 10.1111/vde.70024 (PMC12796996; doi:10.1111/vde.70024)
Supplement: Supplementary file 1 — Appendices S1–S4: vde70024‐sup‐0001‐AppendixS1‐S4.docx. [file VDE-37-34-s001.docx]

**Appendix S1**

**Instruction on how to assess the Content Validity Index (CVI)**

Relevance rating please indicate numbers 1-4:

1 = The question is not relevant to achieve the set objective

2 = The question is somewhat relevant to achieve the set goal (the question would need to be changed to be more relevant)

3 = The question is relevant to achieve the set objective (the question is applicable or requires only minor adjustment)

4 = The question is very relevant to achieve the set goal

Please rate each question individually and if you rate a question less than 3, please indicate why you have chosen this rating.

Based on your rating, we will revise the questions again.

**Formular how to calculate the CVI according to Lynn (1986)**

|  | Number of experts who rated the question as content valid | | | | |
| --- | --- | --- | --- | --- | --- |
| Total number of experts | **2** | **3** | **4** | **5** | **6** |
| 2 | **1,00** |  |  |  |  |
| 3 | 0,67 | **1,00** |  |  |  |
| 4 | 0,50 | 0,75 | **1,00** |  |  |
| 5 | 0,40 | 0,60 | 0,80 | **1,00** |  |
| 6 | 0,33 | 0,50 | 0,67 | **0,83** | **1,00** |

**Appendix S2**


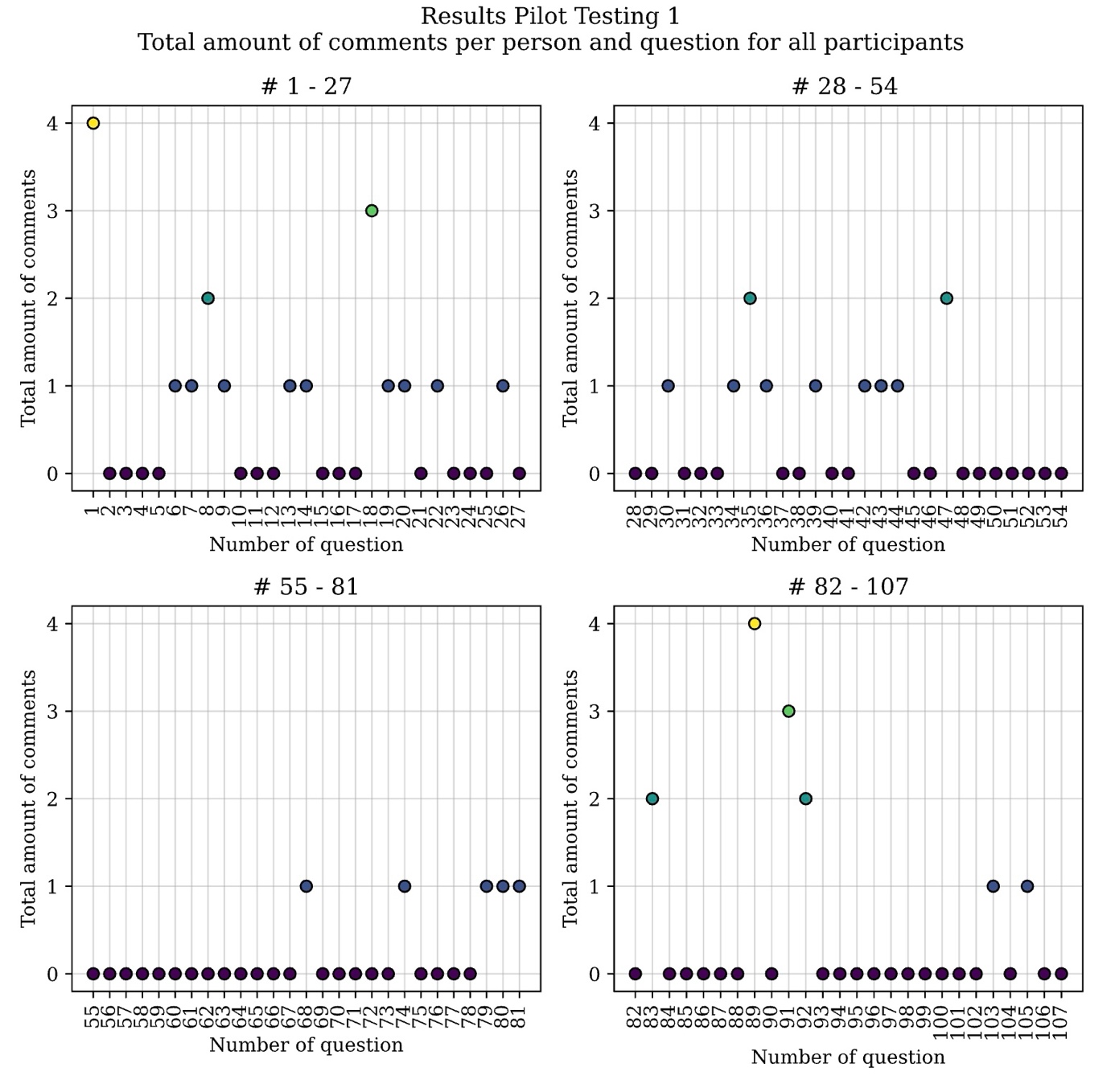


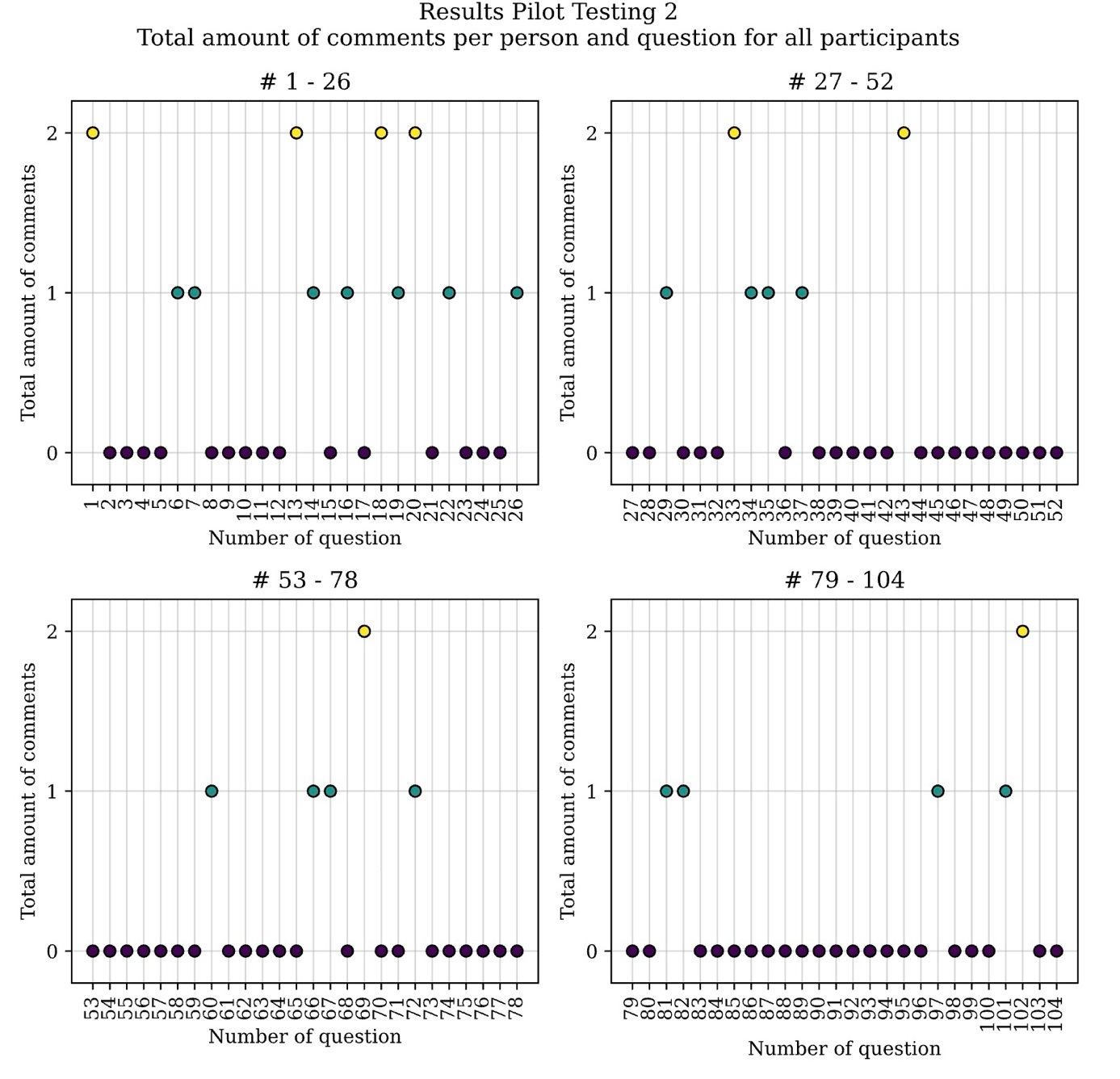


**Appendix S3**

**Example for the rephrasing of a question after pilot-testing round 1**

**Appendix S4**

**Canine dermatological survey**

Thank you for taking part in this survey!

You have received this invitation because you and your dog have previously visited the XXX. This survey aims to gather valuable information about the factors contributing to the development of allergies, which will help us to improve treatment options and our patients’ quality of life.

By answering the following questions, you are actively contributing to this progress.

We guarantee that your information will be treated confidentially.

The survey takes about 30 minutes to complete.

Your answers will be saved, allowing you to at any time.

Please have your dog's pass at hand for the microchip number.

It is not a problem if you answer one or more questions with: “I don't know.” This information is still helpful to us.

There are 104 questions in this survey.

**General information**

**1. Please provide the microchip number of your dog. This helps us to link the data from the survey to your dog’s records in our clinic system. The linkage of information is confined to our veterinary hospital.**

Please write your answer here:

**2. Please provide your dog’s date of birth. If you do not know the exact date, please enter an estimated date of birth.**

Please enter a date:

**3. Please provide your dog’s sex.**

Choose one of the following answers

Please choose **only one** of the following:

- Male
- Female
- Male castrated
- Female spayed

**4. Please provide your dog’s breed (e.g. border collie, French Bulldog, mixed breed)**

Choose one of the following answers

Please choose **only one** of the following:

- Akita
- American Staffordshire terrier
- Australian cattle dog
- Appenzeller Sennenhund
- Australian Shepherd
- beagle
- border collie
- Bernese mountain dog
- Boston terrier
- cocker spaniel
- Chihuahua
- dachshund
- English Bulldog
- French Bulldog
- Great Dane
- German spitz
- German shepherd
- golden retriever
- Irish red setter
- Jack Russell terrier
- Labrador retriever
- pug
- mixed breed
- Rhodesian ridgeback
- toy poodle
- Yorkshire terrier
- other

**Family information**

**5. Was your dog born by caesarean section?**

Choose one of the following answers

Please choose **only one** of the following:

- Yes
- No
- I don't know

**6. Has your dog's mother been diagnosed with an allergic skin disease?**

Choose one of the following answers

Please choose **only one** of the following:

- Yes
- No
- I don't know

**7. Has your dog's father been diagnosed with an allergic skin disease?**

Choose one of the following answers

Please choose **only one** of the following:

- Yes
- No
- I don't know

**8. Have one or more of your dog's littermates been diagnosed with an allergic skin disease?**

Choose one of the following answers

Please choose **only one** of the following:

- Yes
- No
- I don't know

**9. Has a dog from the same breeder as your dog been diagnosed with an allergic skin disease?**

Choose one of the following answers

Please choose **only one** of the following:

- Yes
- No
- I don't know

**Atopic dermatitis**

**10. Has a vet diagnosed your dog with an allergy?**

Choose one of the following answers

Please choose **only one** of the following:

- Yes
- No
- I don't know

**11. Has a specialist (dermatologist) diagnosed your dog with an allergy?**

Choose one of the following answers

Please choose **only one** of the following:

- Yes, a specialist has provided the diagnosis
- No, my private veterinarian has provided the diagnosis
- Other

**12. How old was your dog when the allergy symptoms first appeared?**

Choose one of the following answers

Please choose **only one** of the following:

- < 6 months
- 6-12 months
- 1 year
- 2 years
- 3 years
- 4 years
- Older than 4 years
- I don't know
- Other

**13. Which areas of your dog's skin are affected by the allergy (itching and/or skin lesions)?**


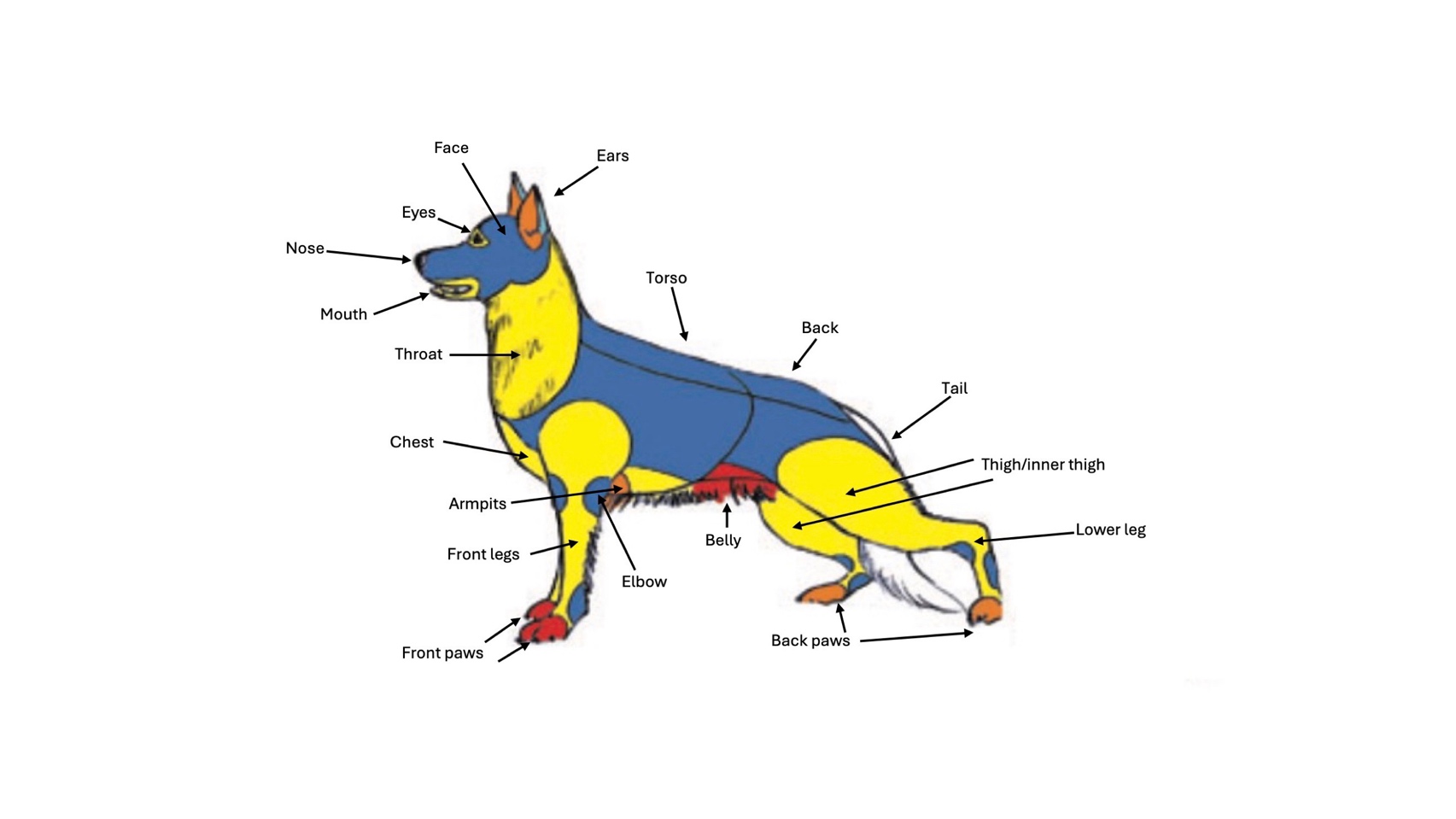
Please choose **all** that apply:

- Face
- Eyes
- Nose
- Mouth
- Throat
- Chest
- Armpits
- Front legs
- Front paws
- Elbow
- Belly
- Back paws
- Lower leg
- Thigh/Inner thigh
- Tail
- Back
- Torso
- Ears
- Other:

**14. Which of the following allergies has a vet already suspected and/or diagnosed in your dog?**

Select all that apply

Please choose **all** that apply:

- Food allergy
- Allergy to certain insect venoms/hypersensitivity to insect bites
- Environmental allergy (mites, pollen, etc.)
- Allergy to infectious agents (malassezia/staphylococci)
- Other:

**15. Has the vet performed an allergy test to investigate the suspected allergy?**

Select all that apply

Please choose **all** that apply:

- Yes, a blood test was performed
- Yes, an intradermal (skin) test was performed
- No, the suspicion was not investigated further by a vet
- Other:

**16. At what age was the allergy test carried out on your dog?**

Select all that apply

Please choose **all** that apply:

- < 1 year
- 1-2 years
- 2-3 years
- 3-4 years
- > 4 years
- I don't know

**17. What was the result of the allergy test?**

Select all that apply

Please choose **all** that apply:

- My dog is allergic to certain foods
- My dog suffers from an environmental allergy (mites, pollen, etc.)
- My dog suffers from an allergy to certain insect venoms/hypersensitivity to insect bites
- My dog suffers from an allergy to infectious agents (malassezia/staphylococci)
- I don't know
- Other:

**18. Based on this scale (numbers 1-10), how would you rate your dog's itching at the moment?**

Choose one of the following answers

Please choose **only one** of the following:


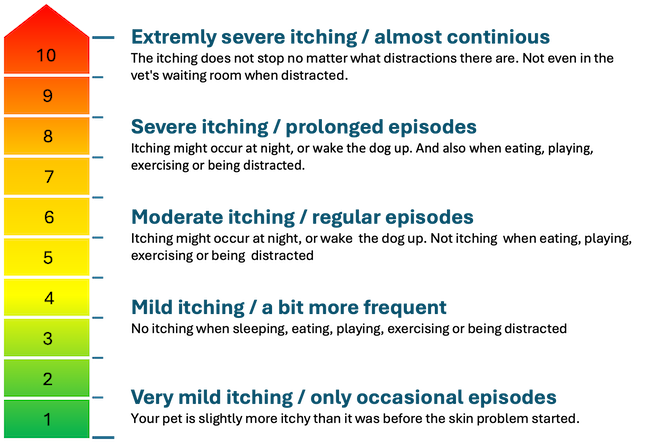


- 1
- 2
- 3
- 4
- 5
- 6
- 7
- 8
- 9
- 10

**19. How does your dog's allergic skin disease manifest itself?**

Choose one of the following answers

Please choose **only one** of the following:

- My dog shows his allergy regularly (all year round)
- My dog goes through phases without allergy symptoms but with relapses
- My dog currently shows no signs of allergy and there have been no relapses so far
- Other

**20. At what time of the year does your dog's allergic skin disease manifest itself?**

Select all that apply

Please choose **all** that apply:

- Spring
- Summer
- Autumn
- Winter
- All year round (continuously)

**21. Which symptoms is your dog currently showing?**

Select all that apply

Please choose **all** that apply:

- Redness
- Crusting
- Excessive hair loss (alopecia)
- Dry skin
- Purulent skin areas
- Erosions (areas of exposed skin)
- Other
- My dog does not currently show any of these symptoms

**22. How long did your dog's last allergic episode last?**

Select all that apply

Please choose **all** that apply:

- Several days WITH treatment
- Several days WITHOUT treatment
- Several weeks WITH treatment
- Several weeks WITHOUT treatment
- Several months WITH treatment
- Several months WITHOUT treatment
- Other:

**Associated diseases**

**23. Has your dog ever had an ear infection (reddening of the ears or head shaking in combination with itching and/or abnormal smell)?**

Choose one of the following answers

Please choose **only one** of the following:

- Yes, the ear infection was treated by a vet with ear drops
- Yes, the vet took a sample from the ears and the ear infection was treated with ear drops
- Yes, but the ear infection was not treated and resolved on its own
- No
- I don't know

**24. How many times a year do your dog's ears become inflamed?**

Please write your answer here:

**25. Has your dog ever been diagnosed with a yeast/fungal/malassezia infection?**

Choose one of the following answers

Please choose **only one** of the following:

- Yes
- No
- I don't know

**26. Which areas were affected by the yeast/fungal/malassezia infection?**

Select all that apply

Please choose **all** that apply:

- Ears
- Skin (any parts of the body surface incl. paws)
- Other:

**27. How often per year are your dog's ears or skin affected by such a yeast/fungal/malassezia infection?**

Please write your answer here:

**28. Did your dog's yeast/fungal/malassezia infection occur in combination with his allergy symptoms?**

Choose one of the following answers

Please choose **only one** of the following:

- Yes
- No
- I don't know

**29. Has your dog ever been diagnosed with a bacterial infection of the skin or ears?**

Choose one of the following answers

Please choose **only one** of the following:

- Yes
- No
- I don't know

**30. Which areas were affected by the bacterial infection?**

Select all that apply

Please choose **all** that apply:

- Ears
- Skin (any parts of the body surface incl. paws)
- Other:

**31. How often per year are the ears or skin affected by the bacterial infection?**

Please write your answer here:

**32. Did the bacterial infection occur in combination with your dog's allergy symptoms?**

Choose one of the following answers

Please choose **only one** of the following:

- Yes
- No
- I don't know

**General hygiene**

**33. When do you deworm your dog?**

Choose one of the following answers

Please choose **only one** of the following:

- In spring
- In summer
- In autumn
- In winter
- My dog does not receive any deworming medication
- I don't know

**34. When is your dog treated for ectoparasites (fleas, ticks, mites etc.)?**

Choose one of the following answers

Please choose **only one** of the following:

- In spring
- In summer
- In autumn
- In winter
- My dog does not receive any medication against ectoparasites
- I don't know

**35. How often do you currently clean your dog's ears?**

Choose one of the following answers

Please choose **only one** of the following:

- Once a day
- Once a week
- Once a month
- Never
- Other

**36. How often do you currently wash your dog with a shampoo?**

Choose one of the following answers

Please choose **only one** of the following:

- Once a day
- Once a week
- Once a month
- Never
- Other

**37. How often do you currently clean your dog’s eyes?**

Choose one of the following answers

Please choose **only one** of the following:

- Once a day
- Once a week
- Once a month
- Never
- Other

**Allergology**

**38. Is your dog currently being desensitized or has your dog already been desensitized in the past?**

Choose one of the following answers

Please choose **only one** of the following:

- Yes
- No
- I don't know

**39. What was/is your dog desensitized to?**

Select all that apply

Please choose **all** that apply:

- Pollen/grasses
- Mites
- Insects: wasps, bees, bumblebees, hornets
- Other:

**40. At what time intervals did the desensitization take place (time intervals of the injections)?**

Select all that apply

Please choose **all** that apply:

- Every week
- Every 2 weeks
- Every 3 weeks
- Every 4 weeks
- Other:

**41.** **How does/did your dog react to the desensitization?**

Choose one of the following answers

Please choose **only one** of the following:

- Complete control of the allergy (no more allergy symptoms)
- Partial improvement of the allergy
- Time-dependent improvement of the allergy (partial/general improvement over time)
- Worsening of the allergy
- No noticeable change
- I don't know

**42. Have you already carried out an elimination diet (change of food/test for food allergy) for your dog's allergy?**

Choose one of the following answers

Please choose **only one** of the following:

- Yes
- No
- I don't know

**43. What food is/was your dog given during the elimination diet? If you do not know this exactly, please tick “Other” and enter the name of the food.**

Select all that apply

Please choose **all** that apply:

- Home-cooked diet
- Commercial hypoallergenic diet
- Commercial diet with hydrolysed protein
- Other

**44. How long did you carry out the elimination diet?**

Choose one of the following answers

Please choose **only one** of the following:

- 1-2 weeks
- 2-3 weeks
- 3-4 weeks
- 4-6 weeks
- 6-8 weeks
- Other

**45. Was your dog only fed the elimination diet during this time? Meaning no additional treats, leftovers from the table etc.?**

Choose one of the following answers

Please choose **only one** of the following:

- Only the elimination diet was fed
- My dog was also given treats, leftovers, etc.
- Other

**The environment your dog was born in**

**46. In which country was your dog born?**

Choose one of the following answers

Please choose **only one** of the following:

- Switzerland
- Germany
- Austria
- Spain
- Italy
- France
- United Kingdom
- Netherlands
- Belgium
- Denmark
- Sweden
- Norway
- Greece
- Turkey
- Rumania
- Bulgaria
- I don't know
- Other

**47. Do you have any information about your dog's life/housing when it was still with the breeder?**

Choose one of the following answers

Please choose **only one** of the following:

- Yes
- No

**48. How would you describe the environment in which your dog was born? What is the location of the breeding facility where you got your dog?**

Choose one of the following answers

Please choose **only one** of the following:

- More urban (>20,000 inhabitants, no contact with farm animals)
- More provincial (>2000 inhabitants, a smaller but lively town/village with both larger buildings and smaller houses, contact with farm animals is possible)
- More rural (<2000 inhabitants, a small town/village with only a few houses and close contact with farm animals)
- Neither of these

**49. What is the approximate altitude of the breeding facility where you originally acquired your dog?**

Choose one of the following answers

Please choose **only one** of the following:

- Less than 1000 meters of altitude
- More than 1000 meters of altitude
- I don't know

**50. In which climate zone is your dog’s breeding facility located?**

**
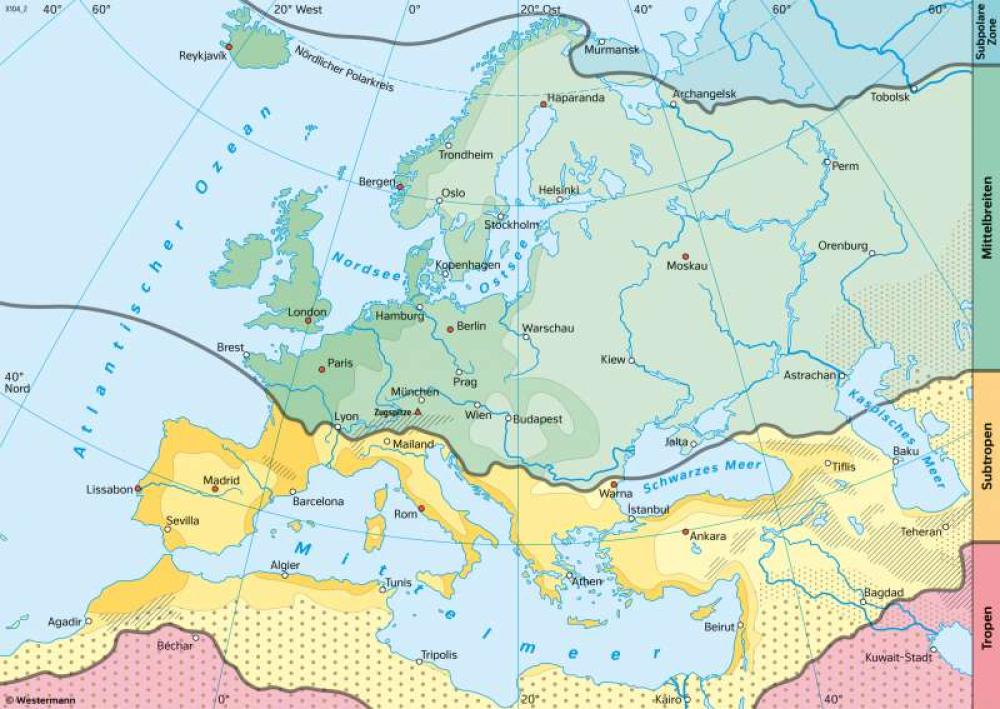
**

Choose one of the following answers

Please choose **only one** of the following:

- Zone A: Mid-latitudes (green)
- Zone B: Mediterranean (yellow)
- I don't know
- Other

**51. Did your dog have contact with other animals at the breeding facility? Did other animals live together with your dog?**

Select all that apply

Please choose **all** that apply:

- Other dogs
- Cats
- Hares
- Rabbits or other rodents
- Exotic animals
- Horses
- Farm animals (cattle etc.)
- I don't know
- Other:

**52. Did your dog have any contact with wild animals at the breeding facility? For example, do wild animals (foxes, deer) have access to the breeder's property?**

Choose one of the following answers

Please choose **only one** of the following:

- Yes
- No
- I don't know

**53. Was your dog only housed in the breeder's house/apartment or could your dog also go outside?**

Choose one of the following answers

Please choose **only one** of the following:

- My dog was only kept in the breeder's house/apartment
- My dog also spent time outside
- I don't know

**54. Are your dog's breeders’ smokers?**

Choose one of the following answers

Please choose **only one** of the following:

- Yes
- No
- I don't know

**55. What type of flooring was in the room/area at the breeding facility where your dog spent most of their time?**

Select all that apply

Please choose **all** that apply:

- Laminate/Hardwood
- Tiles/Stone flooring
- Carpet
- I don't know
- Other:

**56. What type of heating was/is used by the breeder in the room where your dog spent most of their time? If you cannot answer this question, leave the field blank.**

Please write your answer here:

**57. Was there air conditioning in the room where your dog was housed at the breeders’?**

Choose one of the following answers

Please choose **only one** of the following:

- Yes
- No
- I don't know

**58. Do you know if there was a mold problem in the building where your dog was housed at the breeders’?**

Select all that apply

Please choose **all** that apply:

- Yes, there was a problem
- No, there wasn't a problem
- Maybe
- I don't know

**59. Do you know if it was dusty in the building where your dog was kept by the breeder?**

Select all that apply

Please choose **all** that apply:

- Yes, it was
- No, it wasn't
- Maybe
- I don't know

**The environment your dog currently lives in**

**60. Do you currently live in Switzerland with your dog?**

Choose one of the following answers

Please choose **only one** of the following:

- Yes
- No
- Other

**61. If you do not currently live in Switzerland with your dog: Please indicate the country in which you currently live with your dog.**

Please write your answer here:

**62. How would you describe the environment where your dog currently lives? Where is your home located?**

Choose one of the following answers

Please choose **only one** of the following:

- More urban (>20,000 inhabitants, no contact with farm animals)
- More provincial (>2000 inhabitants, a smaller but lively town/village with both larger buildings and smaller houses, contact with farm animals is possible)
- More rural (<2000 inhabitants, a small town/village with only a few houses and close contact with farm animals)
- Neither of these

**63. In which climate zone is your home located?**

**
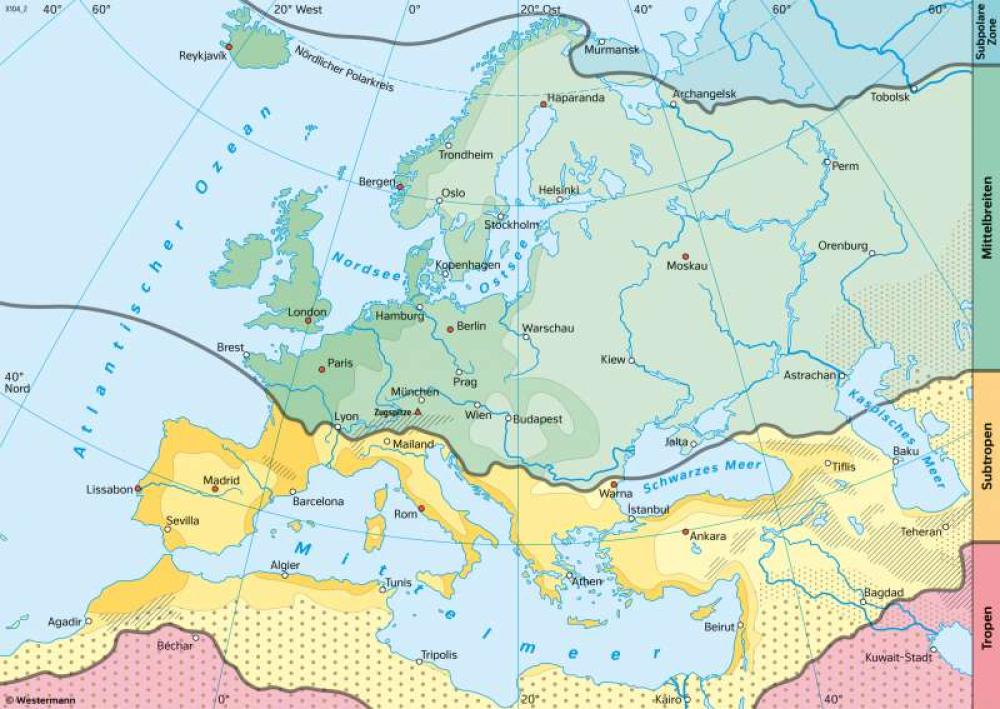
**

Choose one of the following answers

Please choose **only one** of the following:

- Zone A: Mid-latitudes (green)
- Zone B: Mediterranean (yellow)
- I don't know
- Other

**64. Do you live less than 100 kilometres away from the sea?**

Choose one of the following answers

Please choose **only one** of the following:

- Yes
- No
- I don't know

**65. What is the approximate altitude of your house?**

Choose one of the following answers

Please choose **only one** of the following:

- Less than 1000 meters of altitude
- More than 1000 meters of altitude
- I don't know

**66. Does your dog have the opportunity for direct/indirect contact with other animals? If yes, with which animals?**

Select all that apply

Please choose **all** that apply:

- Dogs
- Cats
- Hares
- Rabbits or other rodents
- Exotic animals
- Horses
- Farm animals (cattle etc.)
- My dog has no contact with other animals
- I don't know
- Other:

**67. Does your dog have the possibility of direct/indirect contact with wild animals (foxes, deer, etc.)? For example, can wild animals get onto your property or are you often out walking in the woods?**

Choose one of the following answers

Please choose **only one** of the following:

- Yes
- No
- I don't know

**68. Where does your dog usually sleep?**

Select all that apply

Please choose **all** that apply:

- On the floor (without a blanket)
- On the floor (with a blanket)
- Outside in the yard/garden
- In a dog bed
- On the sofa
- In my bed
- Other:

**69. How many hours a day does your dog spend outside on average?**

Choose one of the following answers

Please choose **only one** of the following:

- 1 hour
- 2 hours
- 3 hours
- 4 hours
- 5 hours
- 6 hours
- 7 hours
- 8 hours
- 9 hours
- 10 hours
- 11 hours
- 12 hours
- 13 hours
- 14 hours
- 15 hours
- 16 hours
- 17 hours
- 18 hours
- 19 hours
- 20 hours
- 21 hours
- 22 hours
- 23 hours
- 24 hours

**70. Is your dog often exposed to cigarette smoke or other types of smoke (e-cigarettes, cigars, pipes)?**

Choose one of the following answers

Please choose **only one** of the following:

- Yes
- No
- I don't know

**71. What type of flooring is laid in the place (home, office, daycare, etc.) where your dog spends most of its time?**

Select all that apply

Please choose **all** that apply:

- Laminate/Hardwood
- Tiles/Stone flooring
- Carpet
- I don't know
- Other:

**72. What type of heating is used in the place (home, office, daycare, etc.) where your dog spends most of its time?**

Please write your answer here:

**73. How often per week do you clean your dog's sleeping area?**

Choose one of the following answers

Please choose **only one** of the following:

- Once
- 2 to 3 times
- 3 to 4 times
- More than 4 times
- Other

**74. Is there air conditioning in the room where your dog spends most of his time?**

Choose one of the following answers

Please choose **only one** of the following:

- Yes
- No
- I don't know

**Feeding**

**75. What food do you feed your dog?**

Select all that apply

Please choose **all** that apply:

- Industrial/commercial food
- BARF (biological, species-appropriate, raw food)
- Self-cooked food
- Other:

**76. Do you feed wet food, dry food or both?**

Choose one of the following answers

Please choose **only one** of the following:

- Dry food
- Wet Food
- Both

**77. What is the main source of protein in your dog's food?**

Select all that apply

Please choose **all** that apply:

- Beef/veal
- Poultry (chicken, duck, turkey)
- Pork
- Lamb
- Venison
- Rabbit
- Goat
- Other:

**78. What is the main source of carbohydrates in your dog's food?**

Select all that apply

Please choose **all** that apply:

- Oats
- Potatoes
- Corn
- Millet
- Wheat
- Other:

**79. When did your dog eat solid food for the first time?**

Choose one of the following answers

Please choose **only one** of the following:

- 2nd week of life
- 3rd week of life
- 4th week of life
- 5th week of life
- 6th week of life
- 7th week of life
- 8th week of life
- Later
- I don't know

**80. Does your dog always get the same food (same protein and carbohydrate) or do you change the food?**

Choose one of the following answers

Please choose **only one** of the following:

- My dog gets the same food every day
- I change the food every few months (approx. every 3 months)
- I change the food regularly (every week)
- My dog eats something different every day
- I don't know

**81. Does your dog get leftovers from the table?**

Choose one of the following answers

Please choose **only one** of the following:

- Yes
- No
- Other

**82. Does your dog get treats?**

Choose one of the following answers

Please choose **only one** of the following:

- Yes
- Yes, but I only use the (normal) dry food as a treat
- No
- Other

**83. What is the quality of the water your dog drinks?**

Choose one of the following answers

Please choose **only one** of the following:

- Hard (calcareous) water
- Soft (deficient in lime) water
- Purchased/filtered water
- I don't know
- Other

**84. How often have you changed your dog's food since you got him from the breeder/animal shelter etc.?**

Choose one of the following answers

Please choose **only one** of the following:

- Never
- Once
- 2 to 3 times
- 3 to 4 times
- More than 4 times
- I don't know
- Other

**85. How old was your dog when you changed the food?**

Select all that apply

Please choose **all** that apply:

- 8 weeks
- 10 weeks
- 12 weeks
- 4-6 months
- 6-8 months
- 8-10 months
- 10-12 months
- Over 1 year
- Over 2 years
- Over 3 years
- I don't know

**86. Do you give your dog additional food supplements (vitamins, plants, etc.)?**

Choose one of the following answers

Please choose **only one** of the following:

- Yes
- No
- I don't know

**87. What supplements do you give your dog?**

Please write your answer here:

**88. Has your dog been given pro- and/or prebiotics in the past?**

Choose one of the following answers

Please choose **only one** of the following:

- Yes
- No
- I don't know

**89. Which pro- and/or prebiotics has your dog been given?**

Please write your answer here:

**Medical history**

**90. Has your dog suffered from another (non-dermatological) disease in the past or is your dog currently suffering from such a disease?**

Choose one of the following answers

Please choose **only one** of the following:

- Yes
- No
- I don't know

**91. From which disease?**

Select all that apply

Please choose **all** that apply:

- Disease of the digestive tract
- Disease of the respiratory tract
- Seasonal conjunctivitis
- Inflammation of the ears
- Other

**92. Which of the following symptoms does/did your dog show more frequently during the illness?**

Select all that apply

Please choose **all** that apply:

- Sneezing
- Coughing
- Asthma symptoms
- Diarrhoea
- Vomiting
- Gurgling or rumbling bowel sounds (borborygmus)
- Farting (flatulence)
- Abdominal pain
- Discomfort of the abdominal area
- Conjunctivitis
- Hives (urticaria)
- Swelling
- Allergic shock
- Other

**93. How old was your dog when the symptoms first appeared?**

Choose one of the following answers

Please choose **only one** of the following:

- < 6 months
- 6-12 months
- 1 year
- 2 years
- 3 years
- 4 years
- Older than 4 years
- I don't know
- Other

**94. Which symptoms is your dog currently showing?**

Select all that apply

Please choose **all** that apply:

- Redness
- Itching
- Crusting
- Excessive hair loss (alopecia)
- Dry skin
- Purulent skin areas
- Erosions (areas of exposed skin)
- Other
- My dog doesn't show any of the above listed symptoms

**95. How often does your dog have diarrhoea within a week?**

Choose one of the following answers

Please choose **only one** of the following:

- My dog usually has no diarrhoea
- Once a week
- More than once a week
- I don't know
- Other

**96. What consistency do most of your dog's faeces have?**

**Please rate the consistency using the following picture.**


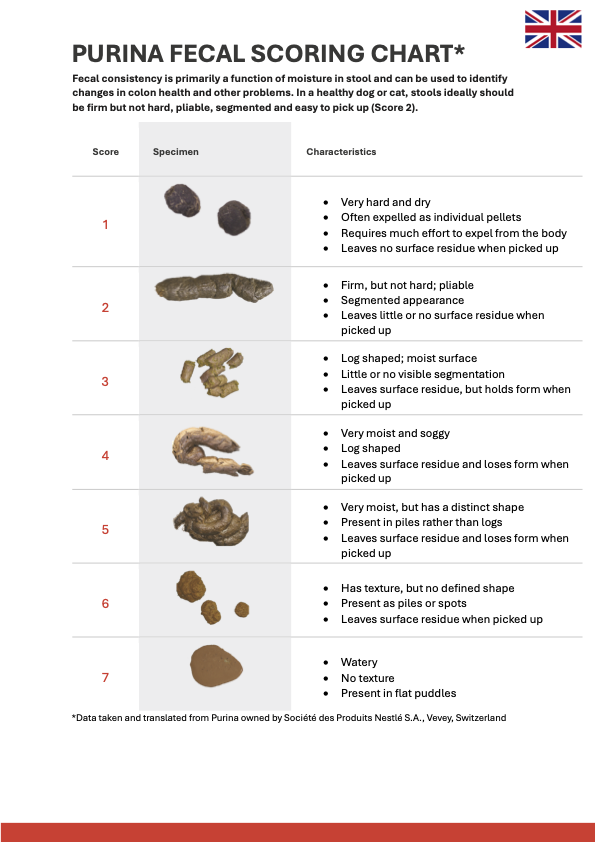


Choose one of the following answers

Please choose **only one** of the following:

- 1
- 2
- 3
- 4
- 5
- 6
- 7
- I don't know
- Other

**97. Did your dog have a severe/acute diarrheal illness within the first year of life?**

Choose one of the following answers

Please choose **only one** of the following:

- Yes
- No
- I don't know

**98. Did your dog have to take antibiotics for more than 2-3 weeks during its first year of life?**

Choose one of the following answers

Please choose **only one** of the following:

- Yes
- No
- I don't know

**Previous treatment**

**99. Has your dog ever been treated with any of the following medications?**

Select all that apply

Please choose **all** that apply:

- Cortisone
- Atopica (Cyclosporin A)
- Apoquel (Oclacitinib)
- Cytopoint (Lokivetmab)
- Antihistamines (z.B. Cetiricin)
- My dog hasn't received any of the above-mentioned medication

**100. What medication is currently being used to treat your dog's allergy?**

Select all that apply

Please choose **all** that apply:

- Shampoos
- Ointment
- Ear drops against inflammation
- Eye drops
- Antibiotics
- Cortisone
- Atopica (Cyclosporin A)
- Apoquel (Oclacitinib)
- Cytopoint (Lokivetmab)
- Antihistamines (e.g. Cetiricin)
- My dog is currently not receiving any medication for his allergy

**Skin and fur**

**101. Does your dog show signs of dry skin (e.g. dandruff)?**

Choose one of the following answers

Please choose **only one** of the following:

- Yes
- No
- I don't know

**102. How would you describe the quality of your dog's fur?**

Select all that apply

Please choose **all** that apply:

- Dull
- Healthy and/or shiny
- Oily
- Discoloured
- I don't know
- Other:

**103. How would you describe the smell of your dog?**

Select all that apply

Please choose **all** that apply:

- Neutral/typical for dogs
- Abnormal smell
- I don't know
- Other:

**104. Is there anything else you would like to mention about your dog's medical history? Please enter your comments in the box below.**

Please write your answer here:
